# Spatio-temporal Rasch analysis of quality of life outcomes in the French general population. Measurement invariance and group comparisons

**DOI:** 10.1186/1471-2288-12-182

**Published:** 2012-11-28

**Authors:** Jean-Benoit Hardouin, Etienne Audureau, Alain Leplège, Joël Coste

**Affiliations:** 1 UPRES EA 4275 (SPHERE) « Biostatistics, Pharmacoepidemiology and Subjective Measures in Health Sciences », University of Nantes, Nantes, France; 2 Plateforme de Biométrie, Direction de la Recherche Clinique, CHU de Nantes, Nantes, France; 3 Nancy-Université, Université Paris-Descartes, Université Metz Paul Verlaine, Research unit APEMAC, EA 4360, Paris, France; 4 Biostatistics and Epidemiology Unit, Assistance Publique-Hôpitaux de Paris, Hôtel Dieu, Paris, France; 5 Department of History and Philosophy of Sciences, University of Paris Diderot - Paris 7, Paris, France

## Abstract

**Background:**

This study aims at analyzing Health related quality of life (HRQoL) data on the French general population between 1995 and 2003 using an Item Response Theory (IRT) model.

**Methods:**

Data concerned 26388 individuals having responded to the SF36 questionnaire in 1995 or in 2003. General Health, Mental Health and Physical Functioning dimensions have been analyzed using a latent regression mixed Partial Credit Model. Differential Item Functioning (DIF) have been searched on each item between age categories, genders, regions of residency, and years of study. Mean and variance of the latent traits have been explained by the same variables, in order to quantify their impact.

**Results:**

Few DIF have been detected between age categories or genders. The analysis shows already known evolutions for HRQoL data: the decrease with age and the differences between genders with worst values for women. We note differences between regions, with better mean value in Paris, in the West or in the South of France, and worst values in the North and in the East. Last, a decrease of the three studied dimensions is noted between 1995 and 2003.

**Conclusions:**

This study using IRT model offers several advantages compared to a classical approach based on scores. First, DIF can be taken into account. More, handling of missing data is easy, because IRT models do not required imputation of missing data. Last, analysis using IRT model is more powerful than analysis based on scores, and allow highlighting a most important number of effects.

## Background

There is considerable interest in measuring and monitoring Health-Related Quality of Life (HRQoL) in general populations. This yields results complementary to traditional mortality and morbidity indicators that could be useful when assessing health disparities or measuring the efficacy of healthcare policies [1-3]. However, monitoring HRQoL through time and space (regions or states within a country, countries) requires the use of measurement instruments which have the crucial property of invariance: the instrument should give the same score for a subject with a given level of HRQoL whatever the time and the place of the measurement. This property is a necessary to assert that differences are genuine and not related to artifacts or other problems in the measurement process.

Item Response Theory (IRT) models, and in particular the models of the Rasch family, are increasingly employed for the validation or the reduction of measurement instruments, and in particular those for HRQoL. These models have, however, been rarely used for analysis of HRQoL datasets [4], even though many measurement instruments have now been validated using this modern measurement theory. Rasch analyses provide unique opportunities to assess simultaneously the invariance of measures and differences between groups. Such analyses also allow the detection and handling of differential item functioning (DIF) [5]: items function differently if they yield different responses patterns across groups of subjects. In addition, the specific objectivity property of the Rasch family models allow obtaining an estimation of the latent concept independent of the answered items of each individual and allows simple handling of missing data, without any imputation being required. These models can be used to perform a latent regression, allowing the explanation of the latent variable by external covariates [6,7]. This latent regression is easy to interpret because, in IRT models, the measured latent trait acquires interval measurement properties [6], i.e. a difference in this latent trait can be interpreted in the same way whatever its level on this latent trait. Last, the homoscedasticity assumption can be relaxed by introducing covariates explaining the value of the variance of the latent trait. This assumption is required when analyzing data with linear models and supposes that the dispersion (variance) of analyzed variable is constant whatever the level of the covariates in the model.

In term of public Health, there is increasing evidence of possible worsening trends in HRQoL in some western countries [1,8,9]. In France, work using Classical Test Theory (CTT) reveals differences through time (1995–2003) and space (administrative regions) [3]. We assessed the independence of these findings from invariance-related issues that could occur in the measurement process. We therefore tested the hypotheses that quality of life differs between regions and has changed between 1995 and 2003 in France; we used a latent regression Rasch model approach, after searching and, if necessary, accounting for DIF for particular covariates (age, gender, region and year of survey) to obtain invariance of measurement for the comparisons between groups of interest in the latent regression model. Here, we present the step-by-step methodology employed and the results for three distinctive dimensions of the MOS-SF36 questionnaire, using data from two large representative surveys of the French population.

## Methods

### Samples

**Figure 1:**
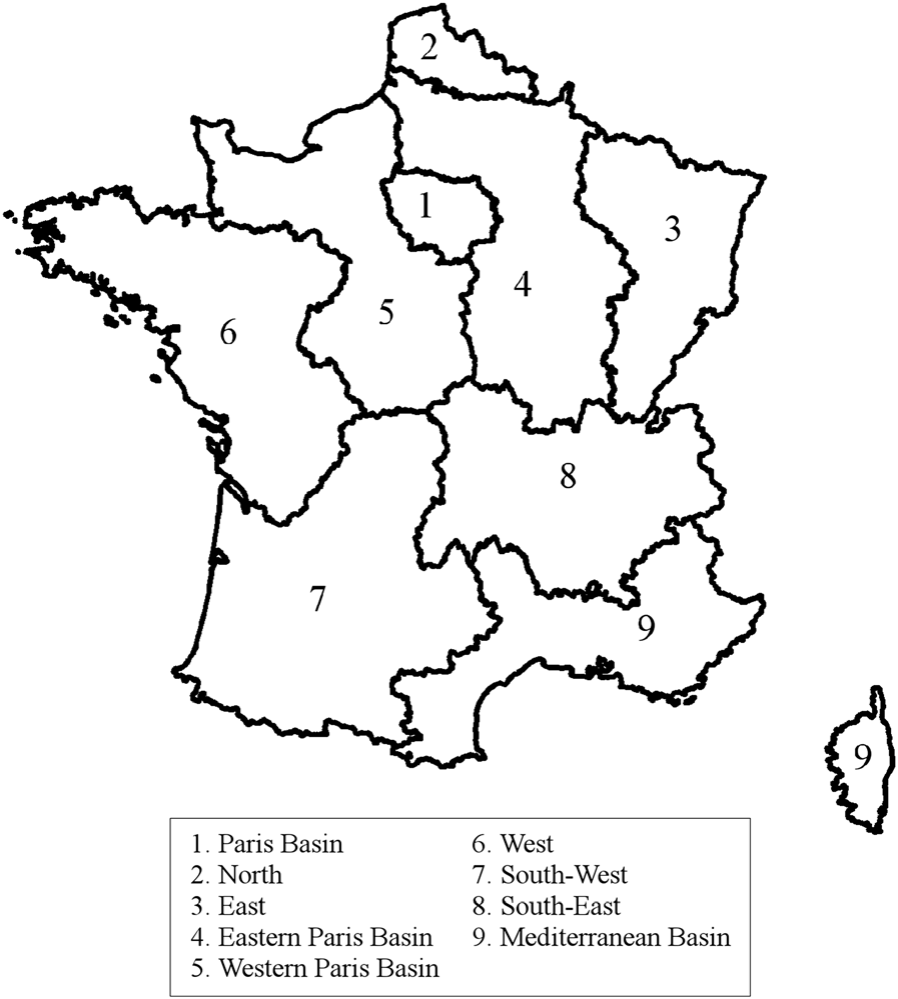
Map of the nine French regions considered in this analysis.

We used two large population-based surveys conducted in France, both representative of the French population [3]. The first was conducted by the SOFRES polling agency in 1995 and included 3656 subjects. The second was conducted by INSEE (French Institute of Statistics and Economic Studies) in 2003 and included 23,018 subjects having completed at least one item of the SF-36 questionnaire. Only subjects aged between 18 and 84 years were retained in the study, giving a total of 26,388 subjects. For each subject, gender, age, and region of residency were recorded (see Figure 1). The age variable was categorized as follows: 18–24, 25–34, 35–44, 45–54, 55–64, 65–74, 75–84 years.

### Questionnaires

For each subject, the responses to the French version of the SF-36 questionnaire [10,11] were available. This questionnaire was developed and validated as part of the International Quality of Life Assessment (IQOLA) project [12]. It is made up of 35 questions divided into eight scales: physical functioning (10 items), role limitations relating to physical health (4 items), bodily pain (2 items), general health perceptions (5 items), vitality (4 items), social functioning (2 items), role limitation relating to mental health (3 items), and mental health (5 items). One additional item assesses changes in health in the last year. The answer to each question was rated on an ordinal scale of between 2 and 6 points. This questionnaire is short and quick to administer (5–10 min) and well-adapted for studies in general populations. It has already been validated with models of the Rasch family [13,14].

### Statistical analyses

The statistical analysis was conducted in three stages as follows: 1. DIF on items was investigated between genders, age categories, region and year of survey; 2. Assumptions of IRT were studied using non parametric Item Response Theory; 3. Partial Credit Models were fitted to data in each dimension of the SF36, and covariates explaining the mean and the variances of the latent trait were tested.

#### Differential Item Functioning detection

A first analysis has been performed to detect DIF for the variables gender, age, region and year of survey. For each combination of gender (2 categories), age (7 categories) and year of survey (2 categories) (28 strata), a Partial Credit Model (PCM) [15] was fitted. This model can be written

(1) $$P\left(Y_{\mathrm{nj}}=y/\theta_{n},\delta_{j}\right)=\frac{\exp\left(y\theta_{n}-\sum_{l=1}^{y}\delta_{\mathrm{jl}}\right)}{\sum_{c=0}^{m_{j}}\exp\left(c\theta_{n}-\sum_{l=1}^{c}\delta_{\mathrm{jl}}\right)}$$

Where Y_nj_ is the response variable of the nth individual (n=1…N) to the jth item (j=1…J), δ_jl_ is the difficulty parameter associated to the lth response category (l=1…m_j_) of the jth item (j=1…J), and θ_n_ (n=1…N) is a set of parameters representing the values of the latent trait for the individuals; δ_jl_ were estimated by the Pairwise Conditional Estimation (PCE) method [16].

In each of the 28 strata, the location associated to each item ( $\frac{1}{m_{j}}\sum_{l=1}^{m_{j}}{\hat{\delta }}_{\mathrm{jl}}$) was estimated. An ANOVA weighted by the sample size used for each model was fitted for each item location by using the gender, the age and the sample as factors, and no interaction. Gender and age effects were studied first. A DIF was considered large if it was significant at 5% and caused a difference greater than 0.1 in the estimation of the mean location between the different levels of a factor.

In a second step, the DIF effects of the region (9 categories) and of the year of survey (2 categories) were evaluated, by estimating the locations of each item for each region and each year (18 models), and by taking into account gender and age effects if DIF had been detected in the previous step.

#### Non parametric Item Response Theory: verification of assumptions

After this DIF detection step, we subjected the data to non parametric Item Response Theory analysis [17]. First, a Monotonely Homogeneous Model of Mokken (MHMM) was fitted to the data. If this model has a good fit, the three fundamental assumptions of the Item Response Theory (IRT) (unidimensionality, local independence and monotonicity) are considered to be verified. This analysis also indicates possibly problematic items and or/subjects. The fit of the model was evaluated using Loevinger’s H coefficients. Three kinds of H coefficients are used to validate the fit of MHMM: H (the coefficient of scalability), H_j_ (the coefficients associated to each item j; j=1,…,J) and H_jk_ (the coefficients associated to each pair of items j,k, j=1…J, k=1…J). A MMHM is considered to have a correct fit if H>0.3, Hj>0.3 and Hjk>0 [17]. The respect of the monotonicity hypothesis was assessed with a specific criterion (Crit_M_); monotonicity is respected if Crit_M_ is below 40 for each item [18].

#### Analysis of the latent trait

For each item, we created a pseudo-item for each level of the variables gender, age category, region and study year which presented a DIF effect [19]. This pseudo-item was given a value equal to the responses of the subjects with this level on the factor and was replaced by a missing value for the other subjects.

A PCM was then fitted on the set of the pseudo-items. In this new model, a random variable is used to estimate the latent trait. This random variable is assumed to be distributed as a normal distribution with mean μ and variance σ^2^. Since an identifiability constraint is required, μ is set to 0. At this step, the PCM was fitted to the data. The sample was very large, and fit tests based on a chi-squared comparison between observed and expected frequencies are highly susceptible to large sample sizes [20]. Therefore, the sample size was artificially set at 500 individuals to evaluate the fit for a sample of moderate size by adjusting proportionally the statistics of the test (that is to say, by multiplying it by 500/N) [21]. This procedure allows avoiding detection of a significant but small and irrelevant gap between the model and the data, as possible with large sample sizes. Indeed, a sample size of only several hundreds of individuals is usually considered as acceptable in order to test the fit of a PCM [20,22,23].

The estimations of the item parameters were set to the estimated values obtained in this model, allowing analysis of the effects of the variables gender, age, region and year of survey on the mean of the latent trait. These variables and their interactions of order 2 were consequently introduced into a latent regression PCM to explain the mean of the latent variable [6]. These variables and interactions were removed one at a time from the model if they were not significant at 5% and if their removal did not increase the value of Akaike’s criterion (AIC) [24].

The variables gender, age, region and year of survey (and their interaction of order 2) were further introduced into the model to explain the variance of the latent trait, and consequently, to account for the heteroscedasticity between groups of subjects. These variables were removed one at a time if they were not significant at 5% and if their omission did not increase the value of Akaike’s criterion (AIC) [24].

The model obtained can be written, as proposed by Boeck and Wilson [6], thus:

(2) $$\begin{array}{l} P\left(Y_{\mathrm{nj}}=y/\Theta_{n}\right)=\frac{\exp\left(y\theta_{n}-\sum_{l=1}^{y}{\hat{\delta }}_{\mathrm{jl}}\right)}{\sum_{c=0}^{m_{j}}\exp\left(c\theta_{n}-\sum_{l=1}^{c}{\hat{\delta }}_{\mathrm{jl}}\right)}\\ \Theta_{n}\sim N\left(\mu_{n},\sigma_{n}^{2}\right) \end{array}$$

$$\mu_{n}=\mu^{*}+X_{n}\beta_{\mu }+\varepsilon_{\mu ,n}$$

$$\sigma_{n}^{2}=\sigma^{2*}+Z_{n}\gamma_{\sigma^{2}}+\varepsilon_{\sigma^{2},n}$$

Where Y_nj_ is the response variable of the nth individual (n=1…N) to the jth item or pseudo-item (j=1…J), Θ_n_ is the latent variable representing the latent trait for the nth individual (n=1…N), ${\hat{\delta }}_{\mathrm{jl}}$ is the estimated difficulty parameter associated with the lth (l=1…m_j_) response category of the jth item or pseudo-item (j=1…J), X_n_ is a set of covariates explaining the mean of the latent variable μ, and β_μ_ is the set of parameters associated with these covariates, Z_n_ is a set of covariates explaining the variance of the latent variable σ^2^, and γσ^2^ is the set of parameters associated with these covariates. μ* and σ^2^* are the estimations of the mean and of the variance in a reference group (men, 18–24 years, Paris basin, year 1995). The ${\hat{\delta }}_{\mathrm{jl}}$ parameters are estimated under the constraint μ=0, such that the global mean of the latent trait is 0.

To evaluate the relative importance of the covariates in explaining the latent trait, we computed the explained variance as follows: let ${\hat{\sigma }}_{0}^{2}$ be the estimated value of the variance of the latent trait in the model without covariates (a model with p_0_ parameters) and ${\hat{\sigma }}^{2}$ the estimated value of the variance of the latent trait in the model with the selected covariates that explain the mean of the latent trait (included in the X_n_ matrix – a model with k parameters); the rate τ of explained variance can be computed as:

(3) $$\tau =1-\frac{\left(n-1-k\right){\hat{\sigma }}^{2}}{\left(n-1-k_{0}\right){\hat{\sigma }}_{0}^{2}}$$

### Studied dimensions

The three most used dimensions of the SF-36 were studied: General Health (GH), Physical Functioning (PF) and Mental Health (MH) (See Additional file 1 for details).

The General Health dimension is composed of five items with five response categories. Three items of this dimension must be inversed [1,3,5]. To analyse the data by IRT, the responses of the item 1 were not weighted and consequently, for each item, the responses were simply coded from 0 to 4.

The Physical Functioning dimension is composed of 10 items with three response categories. None of them need to be inversed and all the response categories were coded from 0 to 2. Three of the sets of items are not locally independent, so recoding of these items was necessary [25] (See Additional file 2 for details).

The Mental Health dimension is composed of five items with six response categories. None of them need to be inversed and all the response categories were coded from 0 to 5.

### Software

The software used included Stata 11 MP [26] (Non parametric IRT analysis [27] and DIF detection), RUMM 2030 (fit of the model) [21] and SAS 9.2 [28] (analysis of the latent trait with the macro-program %Anaqol [29] and the NLMIXED procedure).

## Results

### Descriptive analysis

The common dataset, consisting of data from the two surveys, corresponded to 12272 (46.5%) men and 14116 (53.5%) women. The average age was 46.7 years (SD: 17.0). The distribution of the subjects into regions was: 21% in the Paris basin, 10% in the North, 8% in the East, 15% in the Eastern Paris Basin, 7% in the Western Paris Basin, 11% in the West for, South-8% in the West for, 9% in the South-East for and 11% in the Mediterranean Basin.

### Analysis of the Differential Item Functionning

Table 1 presents the results of the DIF tests for each item of the three dimensions studied, and, for significant DIF, the maximal differences between item locations.

**Table 1:** Maximal difference of the averaged item locations and associated p-value for each item of the three dimensions studied, and for the variables gender, age, region and year of survey

		**Gender**		**Age**		**Region**		**Year of survey**	
		Diff^*^	**p**^**$**^	Diff^*^	**p**^**$**^	Diff^*^	**p**^**$**^	Diff^*^	**p**^**$**^
General Health	GH1	-	0.77	0.70	0.0055	-	0.98	-	0.23
	GH11a	-	0.20	1.01	0.0049	-	0.95	-	0.14
	GH11b	-	0.80	-	0.084	-	0.77	-	0.38
	GH11c	-	0.065	-	0.39	-	0.14	-	0.38
	GH11d	-	0.18	-	0.71	-	0.19	-	0.50
Physical Functioning	PF12	-	0.84	-	0.97	-	0.90	-	0.10
	PF3	0.60	0.005	-	0.26	-	0.51	0.16	0.0264
	PF45	-	0.84	-	0.87	-	0.79	0.17	0.0418
	PF6	-	0.97	0.41	0.0498	-	0.90	-	0.070
	PF789	-	0.56	-	0.067	-	0.81	-	0.14
	PF10	-	0.23	-	0.99	-	0.99	-	0.23
Mental Health	MH1	-	0.89	0.77	0.0003	-	0.86	-	0.89
	MH2	-	0.68	-	0.93	-	0.99	-	0.13
	MH3	-	0.88	-	0.56	-	0.51	-	0.22
	MH4	-	0.35	-	0.71	-	0.99	-	0.95
	MH5	-	0.21	-	0.18	-	0.48	-	0.19

* : maximal difference in absolute value between the estimated locations for each item between two levels of the variable studied.

$ : p-value associated to the maximal difference between the estimated locations for each item between two levels of the variable studied.

The three dimensions studied include 16 items, six of which presented DIF. The first two items of the GH dimension presented DIF between age categories (18–44, 45–64 and 65–84 for GH1; 18–34, 35–64, 65–84 for GH2). The difficulties parameters increased with age for GH1 but decreased with age for GH2.

For the PF dimension, PF3 presented DIF between genders (women considered this item as more difficult than men); PF3 and PF45 presented DIF between year of study (difficulty parameters in 2003 were lesser than these ones in 1995); and PF6 presented DIF between age categories (18–34 and 35–84: difficulty parameters increased with age).

Last, for the MH dimension, only MH1 presented DIF between age categories (18–44, 45–54, 55–64, 65–74 and 75–84 : difficulty parameters decreased with age).

For the following analyses, the items presenting DIF for a variable have been replaced by two or more pseudo-items taking the values of the initial items for the corresponding level of this variable, and missing values for the other levels of this variable. Consequently, 9, 10 and 9 items or pseudo-items were analysed for the dimensions GH, PF and MH, respectively.

### PCM without covariates explaining the latent traits

Non-parametric IRT analysis did not detect any violation of the fundamental assumptions of the IRT for any of the dimensions studied. The fit tests were not significant with a moderate sample size (N=500) for all dimensions (Table 2).

**Table 2:** Fit tests for the modelling of each dimension by a Partial Credit Model (PCM) with the complete sample or with a sample fixed to 500

	**Non parametric IRT analysis**				**Fit of a PCM for the complete sample (n=26388)**			**Fit of a PCM for a fixed, moderate sample size (n=500)**		
	**H**	**Min(H**_**j**_**)**	**Min(H**_**jk**_**)**	**Max(Crit**_**M**_**)**	**χ**^**2**^	**df**	**P**	**χ**^**2**^	**df**	**p**
General Health	0.49*	0.40*	0.31*	−5	1320.42	81	<0.0001	39.36	81	0.99
Physical Functioning	0.76*	0.72*	0.66*	−21	3895.46	94	<0.0001	103.64	94	0.23
Mental Health	0.57*	0.50*	0.33*	−10	2808.10	81	<0.0001	55.95	81	0.98

*: significant at 5%.

Graphical assessment indicated that the latent traits for the three dimensions followed a normal distribution (not shown). For each dimension, the range of values of the difficulty parameters were similar to the range of the latent trait. This demonstrated that the items were adapted to the samples.

### PCM with covariates explaining the latent trait – Construction of the final models

The first step in the construction of the models explaining the latent trait was to recode the variable age such that it could be handled as a quantitative (rather than ordinal) variable. The values were chosen to be coherent with the values of the parameters associated with the dummy variables representing each age category in the preceding analysis.

Tables 3, 4 and 5 present the models obtained for the “General Health”, “Physical Functioning” and “Mental Health” dimensions, respectively. For each model, the constants (for the mean and the variance) represent the mean and the variance of the reference group (men, 18–24 years, Paris Basin, year 1995).

**Table 3:** **Estimation of the parameters associated with the covariates explaining the mean of the latent trait “General Health” (β**_**μ**_**) and with the covariates explaining the variance of the latent trait (γσ**^**2**^**)**

	**Effect**	**Estimate**	**Standard error**	**p-value**
Variables explaining the mean of the latent trait (β_μ_)	Constant (μ*)	0.8238	0.0415	<0.0001
	Women	−0.2121	0.0267	<0.0001
	Survey 2003	−0.1393	0.0413	0.0007
	Ager	−0.0349	0.0019	<0.0001
	East	−0.2039	0.0480	<0.0001
	West	0.1259	0.0344	0.0003
	South-West	−0.0666	0.0301	0.0272
	Ager*Women	0.0049	0.0013	0.0002
	Ager*sample2003	−0.0030	0.0018	0.1047
	Ager* (North, West)	−0.0046	0.0013	0.0004
	Ager* (East)	0.0062	0.0024	0.0096
	Survey 2003*(North, Eastern Paris Basin)	−0.1172	0.0233	<0.0001
	Survey 2003*(Western Paris Basin, South-East)	−0.0675	0.0266	0.0113
Variables explaining the variance of the latent trait (γσ^2^)	Constant (σ^2^*)	1.4130	0.0791	<0.0001
	Survey 2003	−0.1499	0.0837	0.0730
	Ager	−0.0068	0.0034	0.0439
	East	−0.2334	0.0513	<0.0001
	South-West	−0.1254	0.0534	0.0188
	Ager*Survey 2003	0.0068	0.0037	0.0632
Explained variance	Estimated variance without covariates: ${\hat{\sigma }}_{0}^{2}$ (k_0_)	1.4272 (37)		
	Estimated variance with covariates: ${\hat{\sigma }}^{2}$ (k)	1.2406 (50)		
	Rate of explained variances: _τ_	13%		

Ager is a categorical variable taking values 0 for the 18–24, 2 for the 25–34, 12 for the 35–44, 17 for the 45–54, 24 for the 55–64, 32 for the 65–74, and 40 for the 75–84 year-olds.

Women is a variable coded 1 for women and 0 for men.

Survey 2003 is coded 1 for the year 2003 and 0 for year 1995.

Region names are dummy variables coded 1 for the cited region and 0 for other geographical areas.

**Table 4:** **Estimation of the parameters associated with the covariates explaining the mean of the latent trait “Physical Functioning” (β**_**μ**_**) and with the covariates explaining the variance of the latent trait (γσ**^**2**^**)**

	**Effect**	**Estimate**	**Standard Error**	**p-value**
Variables explaining the mean of the latent trait (β_μ_)	Constant (μ*)	3.1131	0.0834	<0.0001
	Women	−1.1154	0.0816	<0.0001
	Survey 2003	−0.2447	0.0512	<0.0001
	Ager	−0.5960	0.0110	<0.0001
	North	−0.2809	0.0696	<0.0001
	Western Paris Basin	−0.1386	0.0723	0.0554
	West, South-West, South-East	0.3284	0.0525	<0.0001
	Women*Ager	0.0667	0.0127	<0.0001
	Ager*(North, Eastern Paris Basin, West, South-West, South-East)	−0.0345	0.0079	<0.0001
Variables explaining the variance of the latent trait (γσ^2^)	Constant (σ^2^*)	8.9934	0.5003	<0.0001
	Women	−2.4176	0.4027	<0.0001
	Survey 2003	3.0001	0.4436	<0.0001
	Ager	−0.5758	0.0650	<0.0001
	Women*Ager	0.1404	0.0533	0.0084
	Survey 2003*Ager	−0.2560	0.0595	<0.0001
Explained variance	Estimated variance without covariates: ${\hat{\sigma }}_{0}^{2}$ (k_0_)	9.3182 (21)		
	Estimated variance with covariates: ${\hat{\sigma }}^{2}$ (k)	6.8119 (30)		
	Rate of explained variances: _τ_	27%		

Ager is a categorical variable taking values 0 for the 18–24, 1 for the 25–34, 2 for the 35–44, 4 for the 45–54, 6 for the 55–64, 8 for the 65–74, and 10 for the 75–84 year-old groups.

Women is a variable coded 1 for women and 0 for men.

Survey 2003 is coded 1 for the year 2003 and 0 for year 1995.

Region names are dummy variables coded 1 for the cited region and 0 for other geographical areas.

**Table 5:** **Estimation of the parameters associated with the covariates explaining the mean of the latent trait “Mental Health” (β**_**μ**_**) and with the covariates explaining the variance of the latent trait (γσ**^**2**^**)**

	**Effect**	**Estimate**	**Standard error**	**p-value**
Variables explaining the mean of the latent trait (β_μ_)	Constant (μ*)	0.5924	0.0313	<0.0001
	Women	−0.4654	0.0186	<0.0001
	Survey 2003	−0.1824	0.0264	<0.0001
	Ager	−0.0936	0.0076	<0.0001
	North, Western Paris Basin	−0.1650	0.0319	<0.0001
	East, Eastern Paris Basin	−0.0872	0.0238	0.0002
	West, South-West	0.1110	0.0319	0.0005
	Ager*(North, West, South-West)	−0.0425	0.0132	0.0005
	Women*(Western Paris Basin)	0.1421	0.0552	0.0101
Variables explaining the variance of the latent trait (γσ^2^)	Constant (σ^2^*)	1.6797	0.0471	<0.0001
	Ager	0.1080	0.0147	<0.0001
	North, Eastern and Western Paris Basins, South-East	−0.1349	0.0530	0.0110
	East, West, South-West	−0.2305	0.0562	<0.0001
	Mediterranean Basin	0.1549	0.0792	0.0505
Explained variance	Estimated variance without covariates: ${\hat{\sigma }}_{0}^{2}$ (k_0_)	1.8377 (46)		
	Estimated variance with covariates: ${\hat{\sigma }}^{2}$ (k)	1.7436 (54)		
	Rate of explained variances: _τ_	5%		

Ager is a categorical variable taking values 0 for the 18–34, 1 for the 35–44, 2 for the 45–64, 3 for the 65–74, and 6 for the 75–84 age groups.

Women is a variable coded 1 for women and 0 for men.

Survey 2003 is coded 1 for the year 2003 and 0 for year 1995.

Region names are dummy variables coded 1 for the cited region and 0 for other geographical areas.

#### General Health

**Figure 2:**
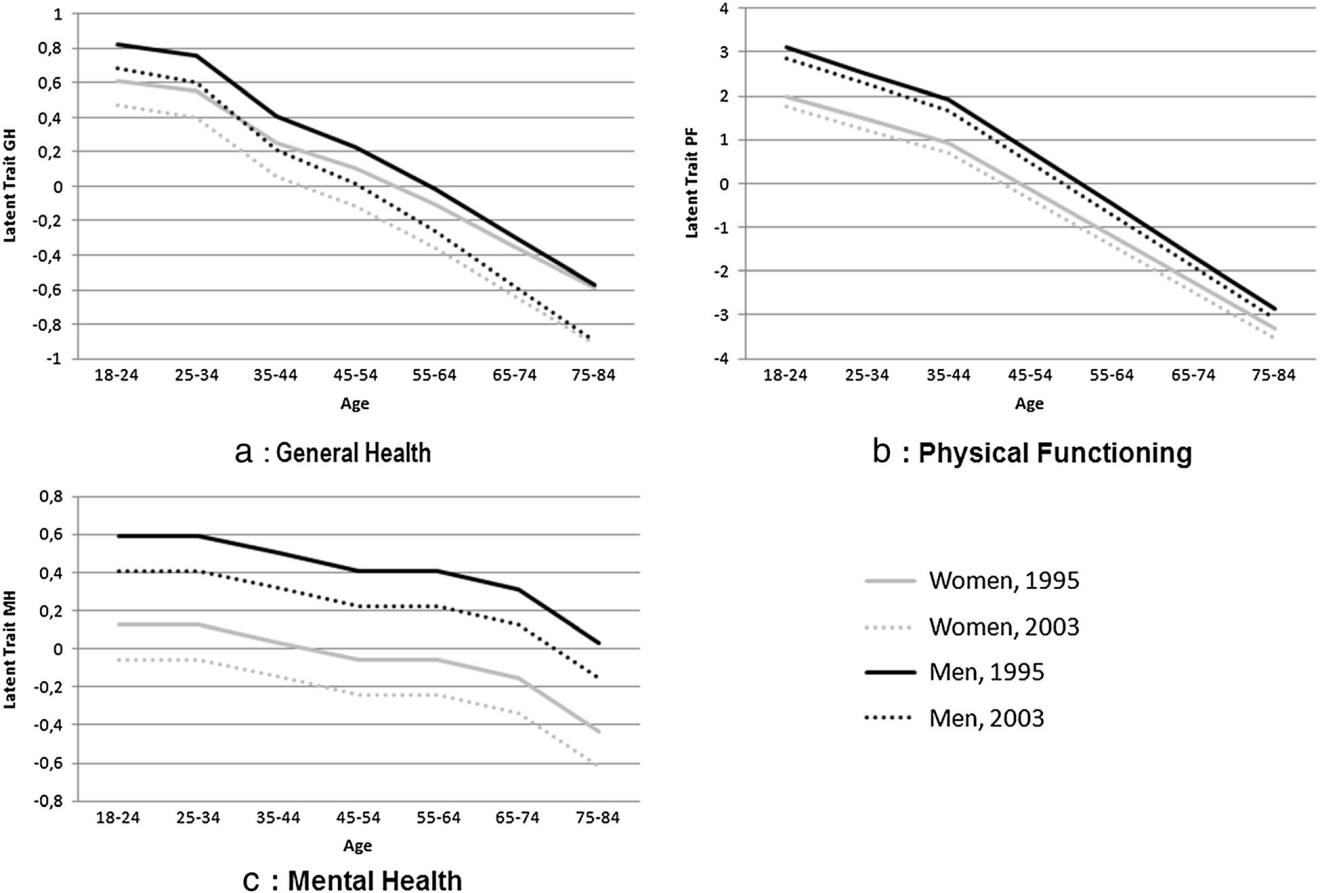
Estimated values of the mean of the latent traits “General Health” (2a), “Physical Functioning” (2b) and “Mental Health” (2c) by gender, survey (1995 and 2003) and age category.

Women gave a lower score than men on the latent trait (for example, -0.21 for the younger age groups). The value of the latent trait decreased with age for men: In 1995, relative to the 18–24 year-old group, the value was −0.07 for the 25–34, -0.42 for the 35–44, -0.59 for the 45–54, -0.84 for the 55–64, -1.12 for the 65–74 and −1.40 for the 75–84 year-old groups. There was a similar trend for women, although the decline in value was lower: In 1995, relative to the 18–24 year-old group, the value was −0.06 for the 25–34, -0.36 for the 35–44, -0.41 for the 45–54, -0.72 for the 55–64, -0.96 for the 65–74 and −1.20 for the 75–84 year-old groups. These trends were stronger in 2003: 75–84 year old men had a value 1.52 lower, and 75–84 year old women a value 1.32 lower than the respective 18–24 year-olds. These trends were significantly stronger in the North and West regions and weaker in the East region. The trends of the mean of the latent trait as a function of the age categories, according to gender and year of survey are given in Figure 2a.

The latent traits in 1995 in the South West region and East region were lower (−0.07 and −0.20, respectively, than in the Paris Basin. Region 6 (West) presented a greater level on the latent trait in 1995 (+0.13) compared to Paris. The decrease between 1995 and 2003 of the mean latent trait was larger in the North region and Eastern Paris Basin, and to a lesser extent, in the Western Paris Basin and South-East region than in the Paris region.

The variance of the latent trait in the reference group (men, 18–24 year-olds, Paris, 1995)was estimated to be 1.4130 . The estimated variance of the latent trait for the 18–24 year-old group in 2003 was lower (average, -0.15). The variance decreased with age in 1995 (0.27 lower for the 75–84 than 18–24 year-olds), but not in 2003 (the age effect (−0.0068) was equal to the parameter associated with the interaction age*sample (0.68)). The variance was lower in the East and South West regions than in the Paris region.

#### Physical Functioning (PF)

Women presented a lower latent trait estimation than men (−1.12 for the 18–24). The level of the latent trait decreased with age for men (relative to the 18–24 year-old group: -0.60 for the 25–34, -1.19 for the 35–44,-2.38 for the 45–54, -3.58 for the 55–64, -4.77 for the 65–74 and −5.96 for the 75–84 year-old groups), and to a lesser extent for women (relative to the 18–24 year-old group: -0.53 for the 25–34, -1.06 for the 35–44,-2.12 for the 45–54, -3.18 for the 55–64, -4.23 for the 65–74 and −5.29 for the 75–84 year-old groups). The latent trait mean decreased between 1995 and 2003 (−0.24).

The latent trait estimation in the West, South West and South-East regions were higher (+0.33), and in North (−0.28) and Western Paris Basin (−0.14) lower than that in Paris. The age trend was more pronounced in the North, Eastern Paris Basin, West, South West and South East regions than in Paris region for men, relative to the 18–24 year-old group, -0.63 for the 25–34, -1.26 for the 35–44,-2.52 for the 45–54, -3.78 for the 55–64, -5.04 for the 65–74 and −6.31 for the 75–84 year-old groups.

The estimated values of the mean of the latent trait “Physical Functioning” as a function of age class, for each gender, in 1995 and 2003, in Paris Basin are given in Figure 2b.

The variance in the reference group (men, 18–24, Paris, 1995) was estimated to be 8.99. The variance was smaller for women (−2.42), and larger in 2003 (+3.00). The variance decreased with age (−0.58 for an increase of 1 in the recoded variable age), and this trend is weaker for women in 1995 (−0.43) and larger in 2003 (−0.73 for men and −0.59 for women).

#### Mental Health (MH)

Women presented a lower latent trait estimation than men (−0.47). This difference is less pronounced in the Centre West than other regions (−0.32). The latent trait estimation decreased with age (relative to the 18–34 year-old group: -0.09 for the 35–44, -0.19 for the 45–64, and −0.28 for the 64–74, -0.56 for the 75–84 year-old groups). The latent trait value decreased between 1995 and 2003 (−0.18).

The latent trait was higher the West and South West regions (+0.11) than in Paris Basin, and was lower in the North and Western Paris Basin regions (−0.17), and to a lesser extent in the East and Eastern Paris Basin regions (−0.09). The age trend was more pronounced in the North, West and South West regions than in the Paris Basin relative to the 18–34 year-old group, -0.13 for the 35–44, -0.27 for the 45–64,-0.41 for the 65–74, and −0.82 for the 75–84 year-old groups.

Figure 2c reports the estimated values of the mean of the latent trait “Mental Health” as a function of the age categories, for men and women in 1995 and 2003, in the Paris Basin.

The variance in the reference group (men, aged 18–34, Paris, 1995) was estimated to be 1.68 and was lower in all the other regions with the exception of region 9 (Mediterranean Basin). The variance increased with the age (+0.11 when the recoded variable age increased of 1).

## Discussion

We describe here a methodology based on the latent regression Partial Credit Model, a model of the Rasch family adapted to polytomous items. Our model allows a latent trait to be explained by external covariates while detecting and handling items presenting DIF, and handling missing values. These last two features are essential to provide measure invariance, because 1) all measures of the latent concept are on the same scale, whatever the group in which the subject is classified, even if perception or functioning of one or several items differ between groups; and 2) the measures of the latent concept are independent of the set of observed items, and can be constructed even in presence of missing responses. As a practical example of the usefulness of this type of method, we applied it to determine whether the worsening trends and regional heterogeneity previously reported in France for HRQoL [3] could be confirmed after obtaining measure invariance.

One major finding of this study was the general decrease of HRQoL in France between 1995 and 2003, as assessed using three dimensions of HRQoL, and after adjustment for region, age and gender variables. The decrease was observed for both genders, all age categories and all the regions considered, without exception. For one dimension (General Health) a significant interaction was found which reinforced the decrease of the latent trait as a function of age category, but did not fully explain the decrease of the latent trait between the two years. Thus, after adjusting for age and gender, we observed a significant decrease of all three quality of life dimensions between 1995 and 2003. The decreases were of moderate size (standardized difference of 0.13 for GH, 0.08 for PF and 0.14 for MH), but it is remarkable that these decreases were systematic for both sexes, all age categories considered and all regions of France (see [3] for a full discussion of this results and limits to interpretation).

We tested for DIF for each item on main covariates. The creation of pseudo items for each level of the variables presenting DIF was used as a straightforward means of accounting for DIF. This is an improvement over the standard use of the scores of the SF36 which does not allow the DIF to be taken into account.

The DIF analysis conducted with the Partial Credit Model also revealed differences for gender or age variables that are easy to interpret: between the two genders, DIF was detected for the item “lift, carry groceries” (PF3); between the age categories, DIF was detected for items relating to general health perception (GH1, GH11a), physical activities (PF6) and work (MH1). Note that no DIF was detected between regions. This result is consistent with the homogeneity of French culture and language across France. By contrast, the DIF detected between the two samples (1995 and 2003) for items PF3 and PF45 is more difficult to interpret, but was, in both cases, only moderate.

Our findings are consistent with established processes concerning differences in quality of life dimensions between age categories and the sexes: HRQoL decreases with age, and is lower for women. Our analysis can be compared with the analysis of the scores as a function of the covariates, using linear regressions [3]. Trends associated with ageing, differences between genders and the decrease between 1995 and 2003 were found in this previous analysis. Concerning the differences between regions, the IRT approach seems to be more powerful, detecting both more and smaller differences between regions. Also, a larger part of variance was explained by the covariates in the IRT approach than in standard linear regression analyses using classical scores (13% vs 11% for GH, 27% vs 20% for PF et 5% vs 3% for MH); this may be due to the detection of a larger number of significant effects associated with regional disparities.

The use of a mixed model of the Rasch family also facilitated handling the problem of missing data in the questionnaire. Indeed, with the specific objectivity of the Rasch family models, the estimations of the parameters linked to the latent trait are consistent whatever the set of items that individuals responded to, whatever the nature (informative or not) of the missing data process [30-32]. In our study, the process of missing data was not at random [33] and therefore could not be ignored. Indeed, if we imputed all the missing data with Personal Mean Score (PMS) (without restriction on the rule of 50% of observed responses), we observe (imputed) mean scores on the General Health dimension of 68.01 for the 22981 individuals (87.1%) without missing data, 65.95 for the 1842 individuals (7.0%) with one or two missing items, and only 56.96 for the 1565 individuals (5.9%) with three or more missing items. The difference between these three means is very significant (p<0.0001) and indicates that the missing data process is very informative. In a classical approach, the 1565 individuals with three or more missing items would not be analysed, and consequently, the latent variable measured by the score would have been overestimated. Also, important assumptions would be made for the 1842 individuals with one or two missing items, although the recommended imputation method (Personal Mean Score) is robust compared to more sophisticated methods such as multiple imputation [34,35].

Our analysis also clearly shows how latent regression models could be used to explain the latent variables by external (predictive) covariates. In a mixed Partial Credit Model, covariates explaining the mean of the latent trait can indeed be simply introduced into the model. This model is more powerful and gives results than are less biased than the method of using a traditional Partial Credit Model to estimate the individual values of the latent trait in a first step, and then in a second step to analyse these individual values with a linear model [36]. Last, in this mixed approach, the homoscedasticity assumption could be relaxed, by explaining the value of the variance of the latent trait by covariates.

Our work raises various issues about the Rasch model approach used. The Partial Credit Models required several assumptions, in particular the three fundamental assumptions of the Item Response Theory (IRT): local independence, monotonicity and unidimensionality. Mokken models gave a good fit to the data, such that we can be confident about the respect of these three assumptions. More, the fit of the Partial Credit Models were satisfactory when Differential Item Functioning (DIF) on some items was taken into account. Previous analyses of the SF36 using Rasch analyses recommend to be vigilant to the problem of local independence of the items of this questionnaire [13], but pointed out the interest of this approach to handle missing data [14].

Three mains limits of this analysis can be noted. First, no data about the health (physical or mental diseases, disabilities…) or the socio-economic context of the individuals (marital status, outcomes, employment…) were available. Consequently, the part of variance explained by the available covariates is limited: only 13% for GH, 27% for PF and 5% for MH. Secondly, the fit of the model have been evaluated only for the model without covariates, because no adapted fit test exists. Last, the two samples (1995 and 2003) are not matched: there are not the same individuals that respond to two dates.

As an extension of this study, a hierarchical model could be envisaged, by considering macro socio-economical variables relevant to the regions; this may improve the part of explained variance of the latent trait. Another potentially informative extension of this work would be the analysis of a new sample, to evaluate the evolution of Health-Related Quality of life in France since 2003.

## Competing interests

The authors declare that they have no competing interests.

## Authors’ contributions

All authors have made substantial contributions to conception and design, analysis and interpretation of data. All authors read and approved the final manuscript.

## Pre-publication history

The pre-publication history for this paper can be accessed here:

http://www.biomedcentral.com/1471-2288/12/182/prepub

## Supplementary Material

Additional file 1 Items of the three studied dimensions of the SF36. Click here for file

Additional file 2 Recoding the Physical Functioning dimension. Click here for file
