# Supplementary material for: Spatio-temporal Rasch analysis of quality of life outcomes in the French general population. Measurement invariance and group comparisons
Source: BMC Med Res Methodol. 2012 Nov 28;12:182. doi: 10.1186/1471-2288-12-182 (PMC3533903; doi:10.1186/1471-2288-12-182)
Supplement: Additional file 1 — Items of the three studied dimensions of the SF36. [file 1471-2288-12-182-S1.pdf]

Appendix 1. list of the items by dimension

General Health

GH1	In general, would you say your health is:	0. Excellent	1. Very Good	2. Good	3. Fair	4. Poor
GH2	How true or false is the following statement? I seem to get sick a little easier than other people.	0. Definitely true	1. Mostly true	2. Don't know	3. Mostly false	4. Definitely false
GH3	How true or false is the following statement? I am as healthy as anybody I know.	0. Definitely true	1. Mostly true	2. Don't know	3. Mostly false	4. Definitely false
GH4	How true or false is the following statement? I expect my health to get worse.	0. Definitely true	1. Mostly true	2. Don't know	3. Mostly false	4. Definitely false
GH5	How true or false is the following statement? My health is excellent.	0. Definitely true	1. Mostly true	2. Don't know	3. Mostly false	4. Definitely false

Physical Functioning

PF1	Does your health now limit you in this activity? If so, how much? Vigorous activities, such as running, lifting heavy objects, participating in strenuous sports.	0. Yes, limited a lot	1. Yes, limited a little	2. No, not limited at all
PF2	Does your health now limit you in this activity? If so, how much? Moderate activities, such as moving a table, pushing a vacuum cleaner, bowling or playing golf.	0. Yes, limited a lot	1. Yes, limited a little	2. No, not limited at all
PF3	Does your health now limit you in this activity? If so, how much? Lifting or carrying groceries.	0. Yes, limited a lot	1. Yes, limited a little	2. No, not limited at all
PF4	Does your health now limit you in this activity? If so, how much? Climbing several flights of stairs.	0. Yes, limited a lot	1. Yes, limited a little	2. No, not limited at all
PF5	Does your health now limit you in this activity? If so, how much? Climbing one flight of stairs.	0. Yes, limited a lot	1. Yes, limited a little	2. No, not limited at all
PF6	Does your health now limit you in this activity? If so, how much? Bending, kneeling, or stooping.	0. Yes, limited a lot	1. Yes, limited a little	2. No, not limited at all
PF7	Does your health now limit you in this activity? If so, how much? Walking more than a mile.	0. Yes, limited a lot	1. Yes, limited a little	2. No, not limited at all
PF8	Does your health now limit you in this activity? If so, how much? Walking several blocks.	0. Yes, limited a lot	1. Yes, limited a little	2. No, not limited at all
PF9	. Does your health now limit you in this activity? If so, how much? Walking one block.	0. Yes, limited a lot	1. Yes, limited a little	2. No, not limited at all
PF10	Does your health now limit you in this activity? If so, how much? Bathing or dressing yourself.	0. Yes, limited a lot	1. Yes, limited a little	2. No, not limited at all

Mental Health

MH1	How much of the time during the past 4 weeks: Have you been a very nervous person?	0. All of the time	1. Most of the time	2. A good bit of the time	3. Some of the time	4. A little of the time	5. None of the time
MH2	How much of the time during the past 4 weeks: Have you felt so down in the dumps that nothing could cheer you up?	0. All of the time	1. Most of the time	2. A good bit of the time	3. Some of the time	4. A little of the time	5. None of the time
MH3	How much of the time during the past 4 weeks: Have you felt calm and peaceful?	0. All of the time	1. Most of the time	2. A good bit of the time	3. Some of the time	4. A little of the time	5. None of the time
MH4	How much of the time during the past 4 weeks: Have you felt downhearted and blue?	0. All of the time	1. Most of the time	2. A good bit of the time	3. Some of the time	4. A little of the time	5. None of the time
MH5	How much of the time during the past 4 weeks: Have you been a happy person?	0. All of the time	1. Most of the time	2. A good bit of the time	3. Some of the time	4. A little of the time	5. None of the time
