# Supplementary material for: Spatio-temporal Rasch analysis of quality of life outcomes in the French general population. Measurement invariance and group comparisons
Source: BMC Med Res Methodol. 2012 Nov 28;12:182. doi: 10.1186/1471-2288-12-182 (PMC3533903; doi:10.1186/1471-2288-12-182)
Supplement: Additional file 2 — Recoding the Physical Functioning dimension. [file 1471-2288-12-182-S2.pdf]

Appendix 2. Recoding the Physical Functioning dimension

In the PF dimension, three sets of items were not locally independent, hence violating one of the three fundamental assumptions of IRT. For each of these three sets, one pseudo item was created to replace each set by a single pseudo-item (19).

For the first set (items PF1 “Limitations for vigorous activity” and PF2 “Limitations for moderate activities”), the pseudo-item PF12 was created with three response categories: 2 if there is no limitation (PF1=2 and PF2=2), 1 if there is a small limitation for major physical effort (PF1=1 and PF2=2) and 0 if there is a limitation for a moderate or major physical effort (PF1=0 or PF2≤1).

For the second set (items PF4 “Climbing several flights” and PF5 “Climbing one flight”), the pseudo-item PF45 was created with three response categories: 2 if there is no limitation (PF4=2 and PF5=2), 1 if there is a small limitation for several steps (PF4=1 and PF5=2) and 0 if there is a limitation for a moderate or major physical effort (PF4=0 or PF5≤1).

For the third set (items PF7 “Walking more than a mile”, PF8 “Walking several blocks” and PF9 “Walking one block”), the pseudo-item PF789 was created with three response categories: 2 if there is no limitation (PF7=2 and PF8=2 and PF5=2), 1 if there is a small limitation for more than 1 kilometre (PF7=1 and PF8=2 and PF9=2) and 0 for the other cases (PF7=0 or PF8≤1 or PF9≤1).
